# Effects of Exercise on Physical Fitness in Older Adults with and Without Severe Cognitive Impairment

**DOI:** 10.3390/bs15030351

**Published:** 2025-03-12

**Authors:** Oliver Ramos-Álvarez, Elkin Eduardo Roldán-Aguilar, Mariano Altamiranda-Saavedra, Juan Carlos Marín, Víctor Arufe-Giráldez

**Affiliations:** 1 Departamento de Educación, Área de Educación Física y Deportiva, Universidad de Cantabria, Los Castros Avenue, 50, 39005 Santander, Spain; 2 Health Economics Research Group—Valdecilla Biomedical Research Institute (IDIVAL), 39011 Santander, Spain; 3 GESTAS Research Group, Politécnico Colombiano Jaime Isaza Cadavid Medellín, Medellin 050022, Colombia; eeroldan@elpoli.edu.co; 4 Technology Applied to Occupational, Equality and Health Research Research Group (TALIONIS), Faculty of Health Sciences, University of A Coruña, de Oza As Xubias University Campus, 15006 A Coruña, Spain; varufe@udc.es; 5 Bioforense Research Group, Tecnológico de Antioquia Institución Universitaria, Medellin 050034, Colombia; maltamiranda2@gmail.com; 6 Tropical Phytotechnics Research Group, Faculty of Agricultural Sciences, Universidad Nacional de Colombia, Medellin 050034, Colombia; juancarlosmo@gmail.com; 7 Education Faculty, University of A Coruña, Elviña University Campus, 15008 A Coruña, Spain

**Keywords:** cognitive decline, exercise, physical fitness, body composition, age

## Abstract

Physical fitness is defined as the ability to perform daily activities efficiently and without excessive fatigue, involving anthropometric variables and physical capacities. This study evaluates the effects of an adapted physical exercise program in older adults with and without severe cognitive impairment (SCI), aiming to compare its effects on blood pressure, anthropometric parameters, and physical fitness. The study included 78 older adults (24 with SCI) who participated in an individualized exercise program for one year, attending at least twice a week. Variables such as body mass index (BMI), body fat percentage (%BF), handgrip strength (HGS), aerobic endurance (6MiWa), balance, coordination, and agility were assessed. The results show significant improvements (*p* < 0.05) in most variables after the intervention, especially in flexibility, walking speed, and coordination. Although adults with SCI started with lower baseline levels, the magnitude of the improvements was similar to that of adults without SCI. However, greater deterioration was observed in handgrip strength and lower limb strength in adults with SCI, highlighting the need for specific interventions for this population. The study concludes that adapted physical exercise improves physical fitness and anthropometric parameters in older adults with and without SCI. It emphasizes the importance of using playful and communicative strategies to ensure the active participation of adults with SCI, promoting their functionality and physical independence.

## 1. Introduction

Physical fitness has been defined as a state of well-being with energy to be able to participate in a wide variety of work, recreational, and daily activities to cope with the demands set by one’s environment without becoming overtired and includes both anthropometric variables and physical capabilities. These demands include activities of daily living (ADLs), which are those necessary for self-care, such as eating and bathing, and instrumental activities of daily living (IADLs), which are necessary for independent living, such as cleaning and cooking (Enriquez-del Castillo et al., 2022; Hilgenkamp et al., 2010). This means having a good level of physical fitness, but this declines with ageing, which increases the risk of sarcopenia and other chronic non-communicable diseases (Pedrero-Chamizo et al., 2015). In addition, with age, cognitive capacity also deteriorates, and neurodegenerative diseases become more frequent, leading to disability, alterations to quality of life, and increased healthcare costs (Hilgenkamp et al., 2010).

Physical fitness is measured according to criteria such as body composition, strength, aerobic capacity, flexibility, agility, coordination, and balance, that is, aspects directly associated with functionality (Garber et al., 2011; J. L. Lobo & Vanegas, 2022). Physical activity is known to improve quality of life and promote physical independence during ageing. In contrast, hypokinetic attitudes are associated with the deterioration of health and increases in health expenditure (Pedrero-Chamizo et al., 2015). One of the ways to counteract rapid deterioration is physical exercise, which, additionally, improves functionality in an individual (Garatachea et al., 2017).

On the other hand, cognitive impairment is defined as a gradual and progressive decline in cognitive abilities over time, commonly associated with ageing or neurodegenerative diseases such as Alzheimer’s (López Miquel & Martí Agustí, 2011). Severe impairment is considered when a score of less than 17 points is obtained in the Mini-Mental State Examination (MMSE) adapted to Spanish by Lobo (A. Lobo et al., 2002).

Adults over 50 years of age with cognitive impairment have lower levels of physical fitness when compared to the general population of the same age. This negatively affects the quality of life and survival in old age of these individuals (Oppewal & Hilgenkamp, 2019). Physical exercise is a tool to counteract these effects (Ballarín-Naya et al., 2021; Colcombe et al., 2006); however, the minimum intensity threshold to obtain physiological adaptations with exercise is between 40 and 59% of maximal reserve consumption (Alemán et al., 2014). Achieving such a stimulus may be challenging in older adults with SCI, as improving fitness requires refined brain control for awareness, motivation, attention to commands, and initiation of specific tasks with respect to muscle recruitment and motor coordination. Therefore, concurrent impairment of brain regions responsible for cognitive and physical performance could be a possible mechanism explaining the association between low fitness and cognitive impairment and the inconsistent fitness improvement outcomes in those with SCI due to inherent variability between individuals and health conditions (Borson, 2010; Oppewal & Hilgenkamp, 2019; Yang et al., 2018).

This requires specific interventions that address barriers such as lack of motivation to exercise in older adults with SCI (Borson, 2010; Oppewal & Hilgenkamp, 2019; Stanish et al., 2006); because of their SCI, they have difficulty understanding exercises and exercise commands, possibly leading to them not performing them with sufficient intensity to elicit physiological adaptations and, therefore, not improving fitness, increasing the risk of chronic non-communicable diseases much more than in those without SCI (Temple et al., 2006). There does not appear to be much scientific evidence on the effect of individualized exercise on changes in fitness, comparing older adults with and without SCI. Although several studies (Christofoletti et al., 2008; Jia et al., 2019; Lamotte et al., 2017; Park et al., 2019) have shown improvements in physical fitness with exercise in patients with severe cognitive impairment, more specifically Alzheimer’s disease, none of them talk about tailored, individualized exercise, let alone comparing the effects of exercise in people with and without SCI. In Colombia, different public entities try to promote physical activity in older adults collectively, but there is no individualized program in these institutions that can meet the needs of older adults in different states of physical and mental health.

Mood is linked to cognitive performance, especially executive function, which, in turn, is linked to motor activity; therefore, the implementation of an adapted exercise program using recreational components could contribute, in some way, to the improvement of physical performance (Herrero Teijón et al., 2024; Verghese et al., 2003). Therefore, it would be important to individualize exercise and adapt it according to individual physical and mental health status, using recreational activities that motivate participants to produce the physiological stimuli that older adults with SCI need to improve their physical fitness in a similar way to those without SCI (Christofoletti et al., 2008; Jia et al., 2019; Lamotte et al., 2017; Park et al., 2019). Therefore, the aim of this study is to evaluate the effects of physical exercise adapted to physical and mental health status on resting blood pressure, anthropometric parameters, and physical fitness in older adults and compare the results obtained between individuals with and without SCI.

## 2. Materials and Methods

### 2.1. Study Design

The study design was a non-randomized, prospective, pre–post clinical trial where physiological measurements were taken before (upon entry—assessment 1), after starting the physical exercise program (intervention), and one year after (assessment 2) having participated continuously in the program, at least twice a week. This research considered two independent variables: physical exercise and the presence of SCI. Dependent variables included blood pressure, anthropometric parameters such as body mass index (BMI), percentage of fat (%F), fat-free mass index (FFMI), abdominal perimeter (PerAb), and various physical capacities, assessed by physical fitness tests. Physical capacities were assessed through different tests: chair seat and reach (ChairSR), manual grip strength (MGS), 30-s chair stand (30SCHS), 6-min walk (6MiWa), eight foot up and go (U&G), right monopodal balance (RMB), left monopodal balance (LMB), and the ‘Soda Pop’ coordination test (SP).

### 2.2. Sample

Out of a total of 112 older adults enrolled in the ‘Solaz bienestar para el adulto’ center (Medellín, Colombia), a total of 78 older adults (24 with SCI and 54 without SCI) who met the inclusion criteria were included in the study. Those participants with cognitive impairments were previously diagnosed by a neurologist, with age-related neurodegenerative cognitive impairment classified as having severe cognitive impairment according to a neurorehabilitation physician for attaining a score of less than 17 points on the Mini-Mental State Examination (MMSE) adapted to Spanish by Lobo (A. Lobo et al., 2002).

### 2.3. Inclusion Criteria

(1) Older adults aged 60 years or older who entered Solaz during the following year. (2) Older adults who had the approval of a doctor specializing in sports medicine to start the physical exercise program. (3) Older adults who remained in the institution for at least one year and who followed the individualized physical exercise program adapted to their cognitive level and state of health at least twice a week. (4) Older adults who were evaluated by the neurorehabilitation specialist to qualify their SCI. (5) Signature of consent by the user, or a responsible relative in the case of those with SCI, could be obtained.

### 2.4. Exclusion Criteria

(1) Individuals who did not meet the inclusion criteria, (2) who voluntarily withdrew or did not undertake the assessments during the intervention year, (3) did not sign the informed consent form, and/or (4) were absent due to illness or other circumstances for more than 2 months during the year of evaluation.

### 2.5. Intervention

In accordance with the medical prescription for exercise issued by a doctor specializing in medicine applied to physical activity and sport, all the older adults were given individualized exercise plans according to their physical and mental state of health. The exercise session lasted one hour and was scheduled at least twice a week, with an initial warm-up phase of 10 min, focused on the mobility of different joints, and 5 min of walking or recumbent cycling at a light intensity of between 10 and 11 according to the perception of effort on the Borg scale (Borg, 1982). This was followed by a main phase of 40 min, where strengthening exercises were performed with an elastic band and body weight at an intensity of between 5 and 6 according to Omni–Ress (Colado et al., 2020) alongside two series of 10 repetitions of exercises targeting the following muscle groups: arm flexors and extensors, pectorals, abductors, shoulder flexors, trunk with abdominals, and lower limbs such as flexors, extensors, abductors and hip adductors, quadriceps, hamstrings, and gastro soleus. Additionally, aerobic endurance was performed at an intensity of perceived exertion, according to the Borg scale, of between 12 and 15, which is equivalent to a percentage of VO_2_max of between 50 and 75% (Katch et al., 2015). The duration was between 20 and 30 min on the Sportrack^®^ treadmill or Proteus^®^ recumbent cycle (Proteus Sports INC, New Taipei, Taiwan). The session ended with a cool down with coordination and balance exercises and, finally, static stretching of the shoulder girdle, quadriceps, hamstrings, and gastro soleus for 20 to 30 s. Older adults with SCI were given the same types of exercises but in a more direct way, with clear commands, which were repeated if necessary, more emotional than rational communication, a playful component, and games to ensure their participation and motivation, all the while maintaining the same quantity and intensities by means of the ‘talk test’ (Colomer & Puig-Ribera, 2022). This is an easy and practical method with which the trainer can monitor the intensity of the effort, since, if the older adult is sweating, blushing, but able to maintain a conversation without being short of breath, it can be assumed, from a physiological point of view, that the individual is exercising at moderate intensity, equivalent to a perception of effort, according to the Borg scale, of between 12 and 15 and a percentage of VO_2_max of between 50 and 75% (Katch et al., 2015).

With regard to the adaptation of exercise to those with comorbidities, the precautions and recommendations made by Penderson et al. (Pedersen & Saltin, 2015) were taken into account (Borg, 1982; Colado et al., 2020).

### 2.6. Procedure

The older adults who were admitted to the center were assessed by a doctor specialized in sports medicine who determined their fitness for exercise. In the same way, the doctor prescribed the exercise according to their physical and mental health condition and carried out the initial physiological tests (physiological variables) (Pre). They were also evaluated by the neurorehabilitation doctor, who classified them from a cognitive point of view and determined whether they had SCI, since those classified at this level are those who have more difficulties understanding the orders given by the instructors to perform physical exercise. All older adults attended the institution at least twice a week and were given physical exercise plans based on the medical prescription and led by a sports professional with a master’s degree in exercise physiology for one year. At the end of the year, the same physiological tests were performed again by the sports medicine doctor (Post).

### 2.7. Physiological Variables

Systolic blood pressure (SBP) and diastolic blood pressure (DBP), in millimeters of mercury (mm Hg): the older adult sat down for at least 10 min and blood pressure was measured on the right arm with a Tycos^®^ blood pressure monitor (Welch Allyn Inc., Skaneateles Falls, NY, USA) and a Lithman^®^ stethoscope (3M, St. Paul, MN, USA). Pressure was taken on two occasions within 5 min of each other, and the average between the two measurements was recorded. The difference between after (Post) and before (Pre) was evaluated and, based on this, the clinical evolution was assessed according to the minimal modifications that the physical activity produced in the blood pressure. The evolution of each patient was classified into three categories: improved (decrease of more than 4 mm Hg), stable (same or no fluctuation of more than 4 mm Hg), or worsened (increase of more than 4 mm Hg) (Chobanian et al., 2003).

### 2.8. Anthropometric Variables

Body mass and height were measured with SECA^®^ digital scales and a SECA^®^ measuring rod (SECA, Hamburg, Germany). The BMI was graded according to the percentiles proposed by Rikli and Jones (Rikli & Jones, 1999). The thickness of skinfolds was measured with a Slinghate^®^ skinfold caliper, with a capacity of 60 mm and a sensitivity of 1 mm, using the technique described by Lohman (Lohman et al., 1988). The subscapular, tricipital, bicipital, and iliocrestal skinfolds were measured and the sum of the four skinfolds and the percentage of total body fat were determined using the logarithmic regressions of the Durnin–Womersley method for the general population (Verghese et al., 2003). In addition, the abdominal perimeter (PerAb) was measured with a Mabes^®^ tape measure, taking as a reference the middle part between the last rib and the iliac crest. During the measurement, the patient was distracted to avoid voluntary contraction of the patient according to the International Society for the Advancement of Kinanthropometry Guidelines (Marfell-Jones et al., 2006). The fat percentage and the patient’s weight were used to calculate the fat weight. Lean weight was obtained by subtracting the fat weight from the total weight. The FFMI was measured to assess the risk of sarcopenia, which was obtained using the following formula:

$$FFMI=FFM÷{height}^{2}\left(m\right)$$

where FFM (fat-free mass) is obtained by subtracting the fat mass (percentage fraction of fat percentage by total weight) from the total weight. Its assessment was made according to the percentiles given by age and gender (Schutz et al., 2002). The PerAb, an anthropometric measure that indicates the fat accumulated in the abdominal viscera, which is responsible for increasing cardiovascular risk, was also assessed (Liu et al., 2021). It was graded according to the World Health Organization (WHO)’s suggestions, as follows: ‘low risk’ if ≤93 cm in men and ≤79 cm in women, ‘increased risk’ if between 94 and 101 cm in men and 80 to 87 cm in women, and ‘high risk’ if ≥102 cm in men and ≥88 cm in women (Aráuz-Hernández et al., 2013).

The clinical evolution across all anthropometric variables was graded as improved (down one category or percentile), stable (remained in the same category or percentile), improved (up at least one category or percentile).

### 2.9. Physical Ability Tests

(1) Manual grip strength (MGS) was measured in kg with a digital hand dynamometer, DIGI-II SH5003^®^, according to the protocol of the American Society of Hand Therapists (Pedersen & Saltin, 2015). The upper body muscular strength was assessed and graded according to the categories presented by Carral et al. (Carral et al., 2000). (2) The ‘Soda Pop’ coordination test (SP) was designed to measure the coordination and mobility of the forearm, wrist, and fingers. The procedure from Carr and Rogerson was used (Carr et al., 2004), and the seconds taken by the subject to perform the test were measured. (3) Right (RMB) and left (LMB) monopodal balance tests were performed on a stable surface, with eyes open, and the time in seconds required to maintain the balance on the surface with one foot (right or left) was measured. Although it is not graded, its evaluation is important to observe the individual’s follow-up, as it assesses both balance and strength, important factors for possible falls (López & Arango, 2015).

The following tests are described in the senior fitness test and were graded according to the percentile for age proposed by Rikli and Jones (Rikli & Jones, 1999). (4) Chair seat and reach test (ChairSR), which measures the centimeters (cm) that are missing (negative number) so that the fingers of the hands touch the tip of the toes, assessing the flexibility of the hamstrings and the lumbosacral spine. (5) The 30-second chair stand (30SCHS) assesses lower body muscular strength and endurance and its unit is the number of times you stand up from the chair, without the help of your hands, over 30 s. (6) The 6-minute walk test (6MiWa), a test that assesses aerobic capacity according to the meters covered in 6 min. (7) The 8 foot up and go (U&G) test measures physical agility and dynamic balance, counting the seconds it takes to walk the distance.

It is important to note that the U&G, FPM, 30SCHS, and ChairSR tests for measuring physical fitness in older adults with SCI have moderate to excellent feasibility and sufficient test–retest reliability (Hilgenkamp et al., 2012).

The clinical evolution of the aforementioned physical capacity variables was graded as follows: worsened, if a category or percentile worsened; stable, if it remained in the same category or percentile; improved, if at least one category or percentile improved.

### 2.10. Statistical Analysis

The relative risk (RR) of an event is the probability of occurrence following exposure to a risk variable compared to the probability of occurrence in a control or reference group and is estimated as the absolute risk with the risk variable divided by the absolute risk in the control group. It is almost always expressed as a ratio with denominator 1 rather than as a percentage (Andrade, 2015).

Multivariate analysis is crucial in medical research, as it allows for the examination of complex relationships among multiple variables which is essential in fields such as epidemiology and clinical research, where diseases and treatments can be influenced by a variety of factors. In addition, multivariate analysis can identify confounding factors and control for them in the analyses, helping to ensure that any observed associations are genuine and not biased by other factors (Tabachnick & Fidell, 2013). Initially, it is advisable to reduce the number of variables as much as possible while capturing as much of the variation in the entire dataset as possible. With regard to techniques for variable selection, two types of analysis were used, i.e., Pearson’s correlation coefficients and a principal component analysis (PCA). The PCA statistical technique is used to reduce the dimensionality of a dataset, preserving as much of its variability as possible. It consists of transforming a set of original variables into a new set of variables, called principal components, which are linear combinations of the original variables (Jolliffe & Cadima, 2016). Additionally, the PCA was used as a tool for data exploration and evaluation of possible correlations between variables (Siswadi et al., 2012). Subsequently, the Wilcoxon signed-rank test was used as a non-parametric test to compare the mean rank of two related samples (without SCI and with SCI) and to determine if there were differences between them.

To observe whether possible confounding variables such as being over 80 years old, having comorbidities, or being physically active twice a week were influencing the differences found in these four variables, the permutational multivariate analysis of variance (PERMANOVA) was used to perform different analyses on the older adults, using the variables %F, BMI, MGS, and 30SCH. The analysis was performed without covariates, with each covariate (ASE, COOM, and AGE), and with combinations of two and three covariates.

Finally, linear discriminant analysis (LDA) was used with the physiological variables measured in older adults with and without SCI. This methodology is useful in several areas, such as medicine, where it has been used to distinguish between pathological and normal groups of subjects based on diagnostic test results (Siswadi et al., 2012). Linear discriminant analysis (LDA) can be a powerful tool for comparing the effects of different treatments between two samples. Its usefulness lies in its ability to maximize the separation between groups based on predictor variables, thus facilitating the identification of distinctive patterns associated with each treatment (Ghadiri et al., 2016).

All statistical analyses, with the exception of RR estimation, were performed using R v.3.5.3 (R Development Core Team, 2008).

### 2.11. Ethical Aspects

The ethical and deontological principles established by the American Psychological Association (American Psychological Association, 2020) were followed in this research. Approval of the research protocol was requested from the Ethics Committee of the Institución Universitaria Politécnico Colombiano Jaime Isaza Cadavid, which granted approval under the code 20610801-201801008579. All participants signed an informed consent form as part of the inclusion criteria (American Psychological Association, 2020).

## 3. Results

### 3.1. Characterization of the Population: Flow Chart and Characterization Table

**Figure 1:**
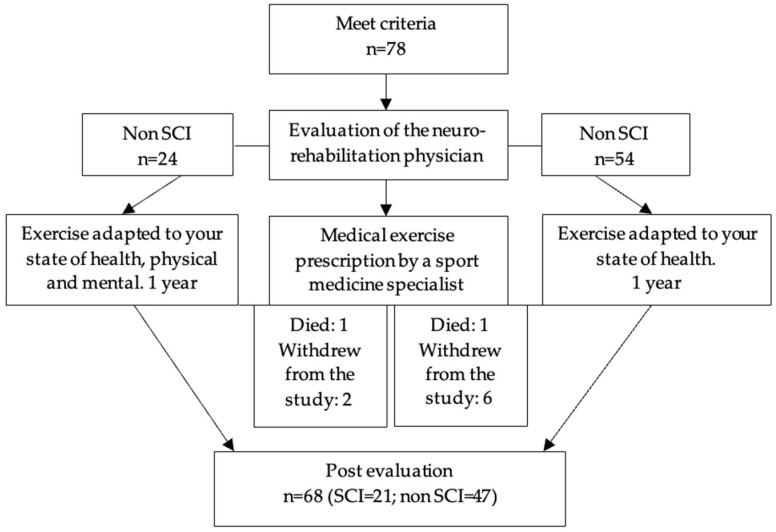
Flowchart of the population inclusion process.

Of the 78 older adults who met the inclusion criteria, the analysis was conducted on a sample of 68 older adults, as eight withdrew and two died. The mean age of the population was 79.2 ± 8 years. Figure 1 shows the flow chart representing the inclusion process of the population.

A total of 69.1% of the population did not have severe cognitive impairment (SCI). The majority were female (63.3%). A proportion of 61.7% exercised twice a week and the rest three or more times a week. Of the latter, 11 (52.4%) had SCI. For the other covariates, the proportions were very similar. Nearly half, 44.1%, had significant comorbidities that could influence physical fitness, but, despite their limitations, the exercise was adapted to their needs, taking the necessary precautions not to produce adverse phenomena and maintaining the intensities and amount of exercise necessary to produce physiological adaptations. Of these, the most important comorbidities were of musculoskeletal origin (22.9%), mainly osteoarthritis of the knee and hip (Table 1).

### 3.2. Results of the Relative Risk of Deterioration by Variables After the Intervention

Of all the variables measured, a significant RR of impairment (*p* < 0.05) was only observed for the variables IMLG and U&G. For IMLG, the RR was 9, with a relative risk increase of 795% and an absolute risk increase of 16.9% for fat-free mass decline in older adults with SCI compared to those without SCI. Although the confidence interval for this variable is wide, it does not cross the reference value 1. For the variable U&G, the RR value was 4.5 with a relative increase in risk of 348% and an absolute increase in risk of 22.2% for gait speed impairment in older adults with SCI compared to those without SCI (Table 2).

### 3.3. Changes in the Variables over Time

Table 3 compares the variables pre- and post-intervention in all the older adults and shows statistically significant improvements (*p* < 0.005) in almost all the variables except for MGS and monopodal balance of both limbs. The variables SBP, PerAbd, ChairSR, and U&G stand out with high significance (*p* < 0.001).

As it can be seen in Table 4, when analyzing the pre-post results between the groups of older adults with and without SCI, physical exercise significantly improved (*p* < 0.05) in both groups: flexibility (ChairSR), aerobic endurance (6MiWa), gait speed (U&G), hand–eye coordination (SP), and right monopodal balance (RMB). This was in spite of the fact that, at baseline, the older adults with SCI started with lower levels (*p* < 0.05) in relation to the first three variables. However, there were no significant differences in these variables when analyzing the differences between the two groups. That is, SCI did not influence the effects of physical exercise on the variables mentioned above compared to the non-SCI group.

On the other hand, there were significant differences in the variables BMI, %F, MGS, and 30SCHS when the analysis was carried out with respect to the differences between the two groups. A greater improvement in the anthropometric variables (BMI and %F) and, on the contrary, a tendency to worsen in the physical fitness variables (MGS and 30SCHS) were documented in the group with SCI compared to the group without SCI. In other words, having SCI did not affect the effects of physical exercise, compared to the non-SCI group, on the aforementioned variables (Table 4).

In multivariate analysis, potential confounding variables such as age, times per week of exercise, and having comorbidities did not influence the results. Overall, interactions with covariates did not show statistical significance (Table 4).

**Figure 2:**
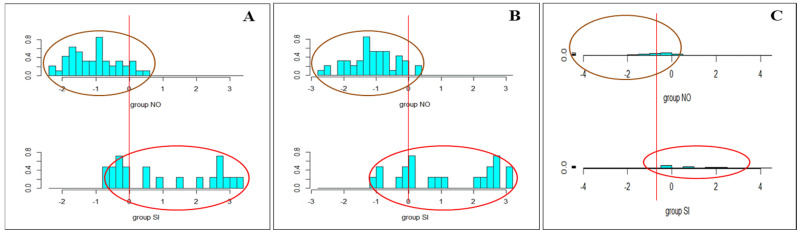
Linear discriminant analysis (LDA) using physiological variables measured in older adults with and without SCI. (**A**) Pre. (**B**) Post. (**C**) Differences between pre- and post-intervention values.

Linear discriminant analysis (LDA) allowed us to identify the variables that contribute the most to the differentiation between the groups. Furthermore, the clear separation observed between the groups suggests the presence of differential effects, attributable to the differential contributions of the measured variables (Figure 2). The LDA obtained from the physiological variables measured in older adults with and without SCI at the ‘pre’ and ‘post’ times reached a classification percentage of 85.29% (Figure 2A,B), obtained with the application of the first two discriminant components that predict the samples according to the group to which they belong. In contrast, the LDA obtained by using the differences between the variables between both times (‘pre’ and ‘post’) showed a decrease in classification accuracy to 80.88%. The implications of the results obtained in these analyses with respect to the objectives set out in this work are discussed below.

## 4. Discussion

Clinical deterioration was found to be greater in older adults with SCI with respect to the variables FFMI, which measures lean mass, and U&G, which measures gait speed. Both are components of physical fitness that are related to survival in older adults with and without SCI (Oppewal & Hilgenkamp, 2019).

A positive correlation was found between 30SCH and 6MiWa (the lower limb strength test with the aerobic endurance test, respectively), a finding which is logical from a physiological point of view, as 30SCH represents lower limb strength which strongly influences walking ability. In the principal component analysis (PCA), correlations of DBP with anthropometric variables such as PerAb (visceral fat) and FFMI (fat-free mass) were found; in another group, the correlation of balance in both lower limbs (RMB, LMB) with lower limb strength (30SCHS), aerobic endurance test (6MiWa), and gait speed (U&G) highly correlated with age. These findings are similar to those found by Wu and Zhao (2021), where different functional tests such as balance, strength, and age correlated with gait speed (Wu & Zhao, 2021).

When the pre–post results of all older adults were compared, the anthropometric parameters improved with exercise after one year across most fitness tests, except for the upper limb strength and balance. Similarly, a study aimed at investigating and comparing the effects of supervised (combined strength and aerobic endurance) and home (guided by a booklet) exercise programs in frail or pre-frail older people for 3 months observed a greater improvement in the supervised exercise group, both in terms of anthropometric parameters and different fitness tests, but, as in this investigation, grip strength improved but did not reach statistical significance and the single leg stance test did not change (Meng et al., 2020).

When analyzing the pre- and post-intervention results obtained in older adults with and without SCI, it was observed that physical exercise led to a significant improvement (*p* < 0.05) in both groups. These improvements included flexibility (chair sit-and-reach test, ChairSR), aerobic endurance (6-minute walk test, 6MiWa), gait speed (up and go test, U&G), hand–eye coordination (stepping plate test, SP), and right single-leg balance (RSLB). However, when comparing the differences between the two groups, no statistically significant variations were found in the magnitude of the observed improvements.

In relation to these findings, recent research (Herrero Teijón et al., 2024; Jolliffe & Cadima, 2016; Romero Ramos et al., 2020) has confirmed the benefits of physical exercise in older adults with SCI. The distinctive aspect of this research lies in the intervention duration, which extended over 12 months, a considerably longer period than previously reported in other studies. This suggests that maintaining physical exercise in the long term could contribute to the sustainability of the observed benefits.

On the other hand, a greater improvement was observed in the SCI group in relation to anthropometric variables, specifically body mass index (BMI) and body composition, reflected in a significant decrease in body fat percentage (%BF). This result could be explained by the characteristics associated with SCI, as older adults with this condition may require assistance in decision making related to their diet, which is a parameter influencing body composition changes. This aspect could not be controlled in this study, as the older adults were not institutionalized and ate at home. Difficulties such as remembering their dietary preferences or understanding available options may allow caregivers to influence them positively, facilitating the provision of a healthier diet (Machado et al., 2023; Meng et al., 2020).

In contrast, the SCI group showed greater deterioration compared to the non-SCI group in two physical fitness variables: handgrip strength (HGS), which indirectly reflects upper limb strength, and lower limb strength, measured through the 30-second chair stand test (30SCHS). These findings are consistent with previous studies (Ghadiri et al., 2016) and could largely be attributed to the decline in muscle mass associated with ageing, which contributes to a decrease in muscle contraction intensity and speed. This process, known as adynamia, is linked to oxidative stress and the natural ageing process. It is plausible that the deterioration observed in muscle strength in older adults with SCI is influenced by underlying physiological and neurological processes related both to ageing and cognitive impairment (Wu & Zhao, 2021). These results highlight the importance of implementing early interventions with physical exercise programs adapted to their physical and cognitive health status to preserve muscle function, improve quality of life, and prevent further functional decline in this population (Chatterji et al., 2015; Hodgson et al., 2021; Hulme & Riddoch, 1993; Leong et al., 2015).

One of the limitations of this study was the relatively small sample size, particularly in the group of older adults with subjective cognitive decline (SCD). Furthermore, it was not possible to control the participants’ diet, as they ate at home, a fact which could have introduced a confounding variable in the results related to body composition.

Nevertheless, the significance of this study lies in the innovative application of an exercise program adapted to the participants’ physical and mental condition. This approach integrated recreational activities and effective and clear communication strategies, achieving significant physiological changes that positively impacted most anthropometric and physical fitness parameters, both in older adults with SCD and those without this condition (Chatterji et al., 2015; Hodgson et al., 2021; Hulme & Riddoch, 1993; Leong et al., 2015; Machado et al., 2023).

## 5. Conclusions

In conclusion, this study demonstrates that intervention with physical exercise adapted to physical and mental health status significantly improves most physical fitness variables and anthropometric parameters in older adults, regardless of the presence of subjective cognitive decline (SCD). Although the SCD group initially exhibited lower levels in some variables, the results indicate that the response to exercise was comparable in both groups, suggesting that SCD does not limit the benefits of physical exercise in terms of physical fitness.

On the other hand, the greater deterioration observed in handgrip strength and lower limb strength in older adults with SCD underlines the need for early intervention to address these limitations. Since muscle mass loss is a key factor in this decline, implementing strategies focused on preserving muscle mass and improving strength is essential to optimize functional capacity and quality of life in this population.

## Figures and Tables

**Table 1:** Frequencies of covariables in both groups.

	Cognitive Impairment		
	SCI	Non-SCI	Total
Total sample	21 (30.9)	47 (69.1)	68 (100)
Gender			
Male	11 (52.4)	14 (29.8)	25 (36.7)
Female	10 (47.6)	33 (70.2)	43 (63.3)
Age group			
<80 years	9 (42.9)	23 (48.9)	32 (47.1)
≥80 years	12 (57.1)	24 (51.1)	36 (52.9)
Weekly frequency of exercise			
Two times per week	10 (47.6)	32 (68.1)	42 (61.7)
Over three times per week	11 (52.4)	15 (31)	26 (38.3)
Comorbidities			
No	11 (52.4)	27 (57.4)	38 (55.9)
Yes	10 (47.6)	20 (42.6)	30 (44.1)
Cardiopulmonary	1 (10.0)	9 (45.0)	6 (9.8)
Neurological	5 (50.0)	2 (10.0)	8 (13.1)
Osteom	4 (40.00)	9 (45.0)	14 (22.9)

Note: the values correspond to the n and, in brackets, to the corresponding percentage.

**Table 2:** Comparison between older adults with and without SCI with respect to the relative risks by variable after the intervention.

Variables	SCI	Impaired		RR	IC 95%
		Yes, n (%)	No, n (%)		
SBP	Yes, n = 21	2 (9.5)	19 (90.5)	0.6	0.1–2.8
	No, n = 47	7 (14.9)	47 (85.1)		
	Total, n = 68	9 (13.2)	59 (86.8)		
DBP	Yes	1 (4.7)	20 (95.3)	0.6	0.1–4.7
	No	4 (8.5)	43 (91.5)		
	Total	5 (7.3)	63 (92.7)		
BMI	Yes	3 (14.3)	18 (85.7)	1.3	0.4– 5.1
	No	5 (10.6)	42 (89.4)		
	Total	8 (11.8)	60 (88.2)		
%F	Yes	1 (4.7)	20 (95.3)	1.1	0.1–11.7
	No	2 (4.3)	45 (95.7)		
	Total	3 (4.4)	65 (95.6)		
FFMI	Yes	4 (19.0)	17 (81.1)	9.0	1.1–75.3 *
	No	1 (2.1)	46 (97.9)		
	Total	5 (7.3)	63 (92.7)		
PerAb	Yes	1 (4.7)	20 (95.3)	0.8	0.1–6.8
	No	3 (6.4)	44 (93.6)		
	Total	4 (5.9)	64 (94.17)		
ChairSR	Yes	1 (4.7)	20 (95.3)	1.1	0.1–11.7
	No	2 (4.3)	45 (95.7)		
	Total	3 (4.4)	65 (95.6)		
MGS	Yes	3 (14.3)	18 (85.7)	1.4	0.4–5.1
	No	5 (10.6)	42 (89.4)		
	Total	8 (11.8)	54 (88.2)		
30SCHS	Yes	4 (19.0)	17 (81.0)	3.0	0.7–12.2
	No	3 (6.4)	44 (93.6)		
	Total	7 (10.3)	61 (89.7)		
6MiWa	Yes	4 (19.0)	17 (81.0)	1.1	0.4–3.3
	No	8 (17.0)	39 (83.0)		
	Total	12 (17.6)	56 (82.4)		
U&G	Yes	6 (28.5)	15 (71.5)	4.5	1.2–16.2 *
	No	3 (6.4)	44 (93.6)		
	Total	9 (13.2)	59 (86.8)		
RMB	Yes	2 (9.5)	19 (90.5)	2.2	0.3–14.8
	No	2 (4.2)	44 (95.8)		
	Total	4 (5.9)	64 (94.1)		
LMB	Yes	4 (19.0)	17 (81.0)	2.2	0.3–14.8
	No	5 (10.6)	44 (89.4)		
	Total	9 (13.2)	59 (86.8)		
SP	Yes	5 (23.8)	16 (76.2)	2.8	0.8–9.4
	No	4 (8.5)	43 (91.5)		
	Total	9 (13.2)	59 (86.8)		

Note: * *p* < 0.05; SCI: severe cognitive impairment; SBP: systolic blood pressure; DBP: diastolic blood pressure; BMI: body mass index; %F: fat percentage; FFMI: fat-free mass index; PerAb: abdominal perimeter; ChairSR: chair seat and reach; MGS: manual grip strength; 30SCHS: 30-second chair stand; 6MiWa: 6-minute walk; U&G: 8 foot up and go; RMB: right monopodal balance; LMB: left monopodal balance; SP: the ‘Soda Pop’ coordination test.

**Table 3:** Changes in medians and interquartile ranges of the physiological variables measured in older adults (total, without SCI grouping, and without SCI).

Variables	Pre	Post
N = 68	Median (Q1, Q3)	Median (Q1, Q3)
SBP ***	120 (110, 130)	110 (110, 130)
DBP *	70 (61, 80)	70 (60, 70)
BMI **	25.8 (23.1, 28.5)	25.6 (22.8, 28.2)
%F ***	35.4 (27.4, 39.4)	33.9 (26.1, 37.9)
FFMI *	17.4 (15.1, 18.4)	17.7 (16.0, 18.5)
PerAb ***	92 (87.2, 99.1)	89.7 (85, 95)
ChairSR ***	−19.9 (−29.0, −8.3)	−14 (−23.0, −4.2)
MGS	18.5 (12.8, 23.3)	17.7 (12.5, 22.9)
30SCHS **	10 (7, 13)	11 (7, 14)
6MiWa *	311 (203, 466)	346 (222, 503)
U&G ***	10.7 (7.8, 22.9)	9.2 (6.7, 16.2)
RMB	3 (0.0, 8.3)	3.5 (0.0, 9.6)
LMB	2.9 (0.0, 8.3)	3 (0.0, 9.0)
SP *	20.8 (16.2, 29.4)	18.5 (14, 26.1)

Note: * *p* < 0.05; ** *p* < 0.01; *** *p* < 0.001; SBP: systolic blood pressure; DBP: diastolic blood pressure; BMI: body mass index; %F: fat percentage; FFMI: fat-free mass index; PerAb: abdominal perimeter; ChairSR: chair seat and reach; MGS: manual grip strength; 30SCHS: 30-second chair stand; 6MiWa: 6-minute walk; U&G: 8 foot up and go; RMB: right monopodal balance; LMB: left monopodal balance; SP: the ‘Soda Pop’ coordination test.

**Table 4:** Changes in pre- and post- intergroup medians and interquartile ranges and differences in effects between the groups with and without SCI.

	Analysis of Differences in Effects Between Groups: Median (Q1, Q3)					
	Non-SCI (n = 47)		SCI (n = 21)			
Variables	Pre	Post	Pre	Post	Non-SCI (n = 47)	SCI (n = 21)
SBP (mm Hg)	120 (110, 130)	110 (110, 120)	120 (110, 120)	110 (108, 110)	−2.0 (−10, 0.0)	−10 (−12, 0.0)
DBP (mm Hg)	70 (60, 80)	70 (60, 70)	70 (65, 80)	66 (60, 70)	0.0 (−10, 0.0)	−10 (−10, 5)
BMI (Kg/m^2^)	26.2 (23.2, 28.7)	26.2 (23.1, 28.5)	24.8 (22.3, 28.3)	23.5 (21.7, 27.2)	−0.1 (−0.7, 0.4)	−0.6 (−1.9, −0.1) *
%F	36.7 (31.6, 39.4)	34.3 (28.2, 38.3)	32.6 (24.4, 39)	27.1 (21.7, 3.5)	−1.2 (−2.4, 0.0)	−2.7 (−5.1, −1.1) *
FFMI (Kg/m^2^)	17.4 (15.8, 18.4)	17.6 (16.1, 18.7)	17.4 8 (16.3, 18.6)	17.8 (15.6, 18.4)	0.2 (−0.2, 0.9)	−0.1 (−0.6, 0.4)
PerAb (cm)	93 (87, 100)	91.3 (85, 95.7)	91.4 (87.5, 98)	88 (54.5, 94)	−2.0 (−5, 0.0)	−3.5 (−6.1, 0.0)
ChairSR (cm)	−15 (−25, −5)	−12.0 (−18, −3) **	−25.0 (−32.5, 14.5)	−23 (−31, −8.5) **	2.0 (0.0, 7)	5 (0.0, 10)
MGS (Kg)	17.9 (12.8, 23.4)	17.9 (13.0, 23.1)	19 (13.8, 22.2)	17.4 (11, 20.2)	0.1 (−1.1, 1.4)	−0.4 (−2.9, 0.1) *
30SCHS (N°)	11 (8, 13)	12 (9, 15)	9 (5, 12)	9 (1.5, 11.5) **	1.0 (0, 0.3)	0 (−2, 1.5) *
6MiWa (m)	354 (218, 483)	410 (247, 578)	281 (198, 411)	288 (119, 443) *	42.9 (0.0, 101)	8 (−38.5, 61.6)
U&G (s)	9.4 (7.2, 17.2)	8 (6, 13) *	15.3 (11.5, 23.9)	15.4 (11, 21.5) ***	−1.1 (−2.4, 0.1)	−0.6 (−4.1, 1.8)
RMB (s)	4 (0.0, 9.8)	4.4 (1, 15) *	1 (0.0, 3.3)	0.0 (0.0, 5.7) **	0.0 (−1, 4.1)	0 (−1, 5.1)
LMB (s)	3.4 (0.0, 9.1)	5 (0, 10)	2.3 (0.0, 5.5)	0.0 (0.0, 5.5) *	0.0 (−1, 3.2)	0 (−2.6, 0.0)
SP (s)	18.3 (14.8, 23.2)	15.6 (13.1, 20.7) ***	48.6 (23.9, 120)	37 (19, 120) ***	−1.7 (−4.1, 0.0)	−6 (2.7, 2.2)

Note: * *p* < 0.05; ** *p* < 0.01; *** *p* < 0.001; SCI: severe cognitive impairment; SBP: systolic blood pressure; DBP: diastolic blood pressure; BMI: body mass index; %F: fat percentage; FFMI: fat-free mass index; PerAb: abdominal perimeter; ChairSR: chair seat and reach; MGS: manual grip strength; 30SCHS: 30-second chair stand; 6MiWa: 6-minute walk; U&G: 8 foot up and go; RMB: right monopodal balance; LMB: left monopodal balance; SP: the ‘Soda Pop’ coordination test.

## Data Availability

Data available on request due to restrictions.
